# How large of a grant size is appropriate? Evidence from the National Natural Science Foundation of China

**DOI:** 10.1371/journal.pone.0264070

**Published:** 2022-02-25

**Authors:** Peixin Duan

**Affiliations:** School of Public Administration and Policy, Shandong University of Finance and Economics, Jinan, China; Sapienza University of Rome, ITALY

## Abstract

Under the current universal trend towards larger grant sizes in research funding systems, we focus on how large of a grant size is appropriate. We study the directional returns to scale (RTS) to assess whether current grant sizes are the most productive. We take the General Program of the National Natural Science Foundation of China (NSFC) as an example and select three samples of physics, geography and management for an empirical study. We find that the optimal input direction and the most productive grant size scale is different for the three disciplines; based on the current grant size, physics should not expand the grant size and team size input, geography should further increase the grant size to improve performance and management should further expand the team size rather than the grant size. In this paper, we demonstrate a new method to calculate the optimal direction, which is the lowest rate of congestion, according to the characteristics of the General Program. Based on these results, we also calculate the most productive scale size. This method has certain value for project management.

## Introduction

Government funding is a key scientific research resource, and research funding systems have recently become more performance-based because such funds come from common taxpayers. One of the most universal trends in research funding systems is increasing grant sizes in all areas, such as Denmark (Bloch *et al.* 2011) [1], Norway (Langfeldt *et al.* 2012) [2], the USA (Arlington, 2007 [3], Katz and Matter 2017 [4]), Canada (Larivière *et al.* 2010 [5], Mongeon *et al.* 2016 [6]) and the European Commission (Matzen 2000) [7]. Increases in grant size can also be found in the National Natural Science Foundation of China (NSFC). For example, the average project grant size for the General Program (*mian-shang xiangmu*), which is the largest program administered by NSFC, increased from 0.18 million yuan in 2001 to 0.72 million yuan in 2020, fourfold increase [8]. A substantial increase in average grant size occurred in 2011, when it doubled from 0.35 million yuan in 2010 to 0.59 million yuan in 2011 [9]. Under the current trend towards larger grants, does greater grant size lead to greater scientific discoveries? Is there an optimal size of research grant? Is the current grant size the most efficient?

A large number of analyses have been conducted concerning the size of research grants [10–13]. Given the nature of research, there are economies of scale and agglomeration effects, implying that scientific productivity is increasing in grant size (Bloch and Sørensen, 2015 [11], Tomas *et al.* 2018 [13]). Research funding concentration can result in the type of cumulative influence known as the ‘the Matthew effect’ (Merton 1968 [14]; 1988 [15]). Some suggest that the performance of acquiring additional funds increases with grant size (OECD 2014 [10]; Bloch and Sørensen 2015 [11]; Bloch *et al.* 2016 [16], Ida and Fukuzawa 2013 [17]). Large grants and concentration is also promoted as a means to avoid the dilution of resources and as a necessary precondition for efficiency in terms of larger scientific outputs (Hicks and Katz 2011 [18], Vaesen and Katzav 2017 [19]) For example, an OECD report (OECD 2014 [10]) suggests that the amount of funding correlates positively with the level of diversity in interdisciplinary collaboration and that collaborations lead to new types of research and possibilities to pursue high-impact, high-risk, and long-term goals. According to Bloch and Sørensen (2015) [11], large grants or centers facilitating larger grants is an important factor in stimulating interaction and mutual learning projects that are otherwise not possible. Large-scale research funding leads to an increase in the number of papers in some fields and an increase in citation counts in other fields (Ida T. and Fukuzawa, 2013 [17]).

Despite the trend towards larger grants, there is no clear evidence that larger grants are associated with better research performance. Recent studies have questioned whether larger grants have the desired impact on research performance and pointed to possible unintended negative effects of larger research grants (Fortin and Currie, 2013 [20]; Bloch and Sørensen, 2014 [11]; Bloch *et al.*, 2016 [16], Mongeon *et al.*, 2016 [6], Vaesen and Katzav, 2017 [19]). Fortin and Currie (2013) [20] conclude that scientific impact (as reflected by publications) is only weakly limited by funding and that greater productivity is not strongly related to greater funding. Gök et al. (2016) [21] empirically analyzed six small advanced European economies and found that lager grant size is negatively related to citation impact. Mongeon *et al.* (2016) [6] find that increasing research funding yields decreasing marginal returns with respect to research output and citation impact (including top 10% most cited) in health research. Aagaard *et al.* (2020) [22] reviewed the key findings from 20 years of empirical research on the relation between the size of research grants and scientific performance and finds stagnant or diminishing returns to scale for the relationship between grant size and research performance. Dimke *et al.* (2019) [23] surveyed Danish scientists about their ideal research grant: most surveyed scientists preferred small- or medium-sized grants to pursue their ideas and advance their scientific careers. Osório and Bornmann (2021) [24] suggest that the research of small teams is more likely to be disruptive than that of large teams.

Thus, it is unclear whether larger grant sizes promote research up to now? In the most universal trend towards larger programs, a general result for research size appears is that a critical mass threshold for grant sizes exists, but with no empirical evidence of returns to scale (RTS) (Bloch *et al.*, 2016) [16]. Therefore, we focus on whether current grant sizes are the most productive by studying the RTS of research projects. By means of the DEA method, we study the directional RTS according to the characteristics of the unequal proportion of research project input. First, based on the grants of the NSFC’s General Program, this paper studies the change in directional RTS in the presence of congestion and constructs a directional DEA model under congestion. Then, we develop an optimal input direction calculation method instead of randdomly selecting the direction to calculate RTS. Second, we select three samples as an example for empirical analysis and determine the optimal input direction. Third, on the basis of the above results, the most productive scale size is determined in the optimal input direction. This paper also explores whether there are differences in the investment ranges of different disciplines to provide a useful reference for future funding strategies.

## Directional RTS in DEA

RTS, which originates from economics and is related to the concept of elasticity, refers to the relative change rate of an output when all the inputs change proportionally. RTS is useful for a manager when he or she wants to decide whether to increase or decrease the size of organization; in other words, RTS can provide useful information on the optimal size of an organization. Frisch(1965) [25] noted that in classical economics, the analysis of RTS is mainly for the production function of the single output technology. In actual production, the real production function is often difficult to obtain, especially in the case of multi-input/multi-output production.

Data Envelopment Analysis (DEA) is a “data-oriented” approach for evaluating the performance of a set of peer entities called Decision-Making Units (DMUs), which convert multiple inputs into multiple outputs (Cooper *et al.*, 2011 [26]). And the frontier of DEA can effectively estimate the real production function, so many problems related to production function in economics can be solved in DEA method such as minimum cost, maximum benefit and RTS. Banker *et al.* (1984) [27] first introduced RTS from the classical economics into the DEA framework. Since then, the issues of RTS in DEAdeveloped the radial measure CCR model (Banker and Thrall, 1992 [28]), BCC model (Banker *et al.*, 1996 [29]) and FGL model (Färe *et al.*, 1985 [30], 1994 [31]) to determine the RTS of DMUs (for a review, Banker *et al.* 2011 [32]). Afterwards, Zhu (2001) [33] and others proposed a DEA model based on nonradial measure for RTS (Chen, 2003 [34], Tone, 2003 [35], Soleimani *et al.*, 2006 [36], Sueyoshi and Sekitanni, 2007 [37], Lee *et al.*, 2014 [38], Taleb *et al.*, 2022 [39], Kao, 2022 [40]), making up for the defect of missing slack variables in the radial measurement. To offer a stronger quantitative characterization of RTS than the qualitative approach, most DEA research efforts in developing the quantitative approach to measure scale elasticity (Golany and Yu, 1997 [41], Førsund and Hjalmarsson, 2004 [42], Podinovski and Forsund, 2010 [43], Schubert and Yang, 2016 [44], Natesan and Marathe, 2017 [45]).

The above research is based on the definition of RTS by Banker *et al.* (1984) [27] under the premise of the classic DEA assumption that the input and output change proportionally. However, in practice, especially in scientific research, inputs usually do not change proportionally. For example, the funding size of the NSFC General Program has been increased multiplicatively. Meanwhile, due to limited time and energy, team size may not increase proportionally: it is limited to the scale of trained students (especially doctoral students) and the time and energy of scientific researchers. Fukuyama (2003) [46] proposed a directional technology scale elasticity formula, in which Farrell input-output scale elasticity can be included as a special case. Directional distance function was proposed to measure RTS and efficiency (Portela *et al.*, 2004 [47], Tavana *et al.*, 2018 [48], Sun *et al.*, 2019 [49], Kerstens and Van, 2018 [50]). Balk *et al.*, (2015) [51] measured scale elasticity as a directional derivative and calculated the scale elasticity along any direction. In addition to the aforementioned studies, Yang (2012) [52] introduced the concepts of directional RTS and directional scale elasticity into the DEA model (abbreviated as Yang’s method hereafter). Yang *et al.* (2014) [53] and Yang and Liu (2017) [54] provided a procedure for estimating directional RTS of strongly efficient DMUs based on the finite difference method (FDM) (Rosen *et al.*, 1998 [55], Golany and Yu, 1997 [41]). Jahanshahloo and Talebian (2017) [56] based on Yang’s method investigate the directional RTS in a new perspective the defining hyperplanes of VRS production. Instead of Yang’s method, Ren *et al.* (2021) [57] provide a straightforward derivation for the formula of the directional scale elasticity and accordingly construct two auxiliary linear programs to obtain the exact values of the right- and left-hand directional scale elasticity.

In the following, we construct the directional RTS DEA model and determine the PPS set under Yang’s method frame. Instead of randomly selecting the direction to calculate RTS with Yang’s method, we innovate an optimal input direction calculation method. And then the most productive scale size is determined.

### PPS of directional RTS and the determination of RTS

In view of the nonproportional input change in scientific activity, we investigate the directional RTS in the way introduced by Yang et al. [53]. Input and output constitute a set of production possibilities (*X*_*j*_, *Y*_*j*_), *j* = 1, …, *n*, where the input vector *X*_*j*_ can produce output vector *Y*_*j*_, and the PPS satisfies:

$$\begin{array}{c} P_{BCC}\left(X,Y\right)=\{\left(X,Y\right)|X\geq \sum_{j=1}^{n}\lambda_{j}X_{j},Y\leq \sum_{j=1}^{n}\lambda_{j}Y_{j},\sum_{j=1}^{n}\lambda_{j}=1,\lambda_{j}\geq 0,j=1,\ldots ,n\}. \end{array}$$ (1)

Assume $DMU\left(X_{0},Y_{0}\right)\in PPS,X_{0}\in R_{+}^{m},Y_{0}\in R_{+}^{s}$ and *β*(*t*) = max{*β*|(Ω_*t*_ *X*_0_, Φ_*β*_ *Y*_0_) ∈ *PPS*, *t* ≠ 0}, where Ω_*t*_ = *diag*{1 + *ω*_1_*t*, …, 1 + *ω*_*m*_ *t*}, Φ = *diag* 1 + *δ*_1_ *β*, …, 1 + *δ*_*s*_ *β*, (*ω*_1_, …, *ω*_*m*_)^*T*^ and (*δ*_1_, …, *δ*_*s*_)^*T*^ represent input and output directions, respectively, and satisfy $\sum_{i=1}^{m}\omega_{i}=m$, $\sum_{r=1}^{s}\delta_{r}=s$, where *t* and *β* represent the change in input and output, respectively. If

$$\begin{array}{c} \rho^{-}=\underset{t\to 0^{-}}{\text{lim}}\frac{\beta (t)}{t},\rho^{+}=\underset{t\to 0^{+}}{\text{lim}}\frac{\beta (t)}{t} \end{array}$$ (2)

then the directional RTS of *DMU*(*X*_0_, *Y*_0_) is as follows: if *ρ*^−^ > 1 (*ρ*^+^ > 1) holds, increasing RTS of *DMU*(*X*_0_, *Y*_0_) prevails in the direction of (*ω*_1_, …, *ω*_*m*_)^*T*^ and (*δ*_1_, …, *δ*_*s*_)^*T*^; if *ρ*^−^ = 1 (*ρ*^+^ = 1) holds, constant RTS of *DMU*(*X*_0_, *Y*_0_) prevails in the direction of (*ω*_1_, …, *ω*_*m*_)^*T*^ and (*δ*_1_, …, *δ*_*s*_)^*T*^; if *ρ*^−^ < 1 (*ρ*^+^ < 1) holds, decreasing RTS of *DMU*(*X*_0_, *Y*_0_) prevails in the direction of (*ω*_1_, …, *ω*_*m*_)^*T*^ and (*δ*_1_, …, *δ*_*s*_)^*T*^.

### Directional DEA model and the determination of RTS under congestion

Because the research program input scale, such as team size and grant size, does not change proportionally, the output of NSFC will not always increase with an increase in grant size input, and sometimes even output will be congested. Congestion is a special return to scale (Wei and Yan, 2004) [58] and in this paper it means that excessive amounts of an input lead to a reduction in the output (Khodabakhshi, *et al.* (2014) [59]). Thus, on the basis of the directional RTS introduced by Yang *et al.* [53] and the congestion research of Wei and Yan *et al.* [58], we investigate the directional RTS under congestion. The congestion effect exists in DMU, so the PPS may satisfy (Wei and Yan, 2004) [58]

$$\begin{array}{c} P_{convex}\left(X,Y\right)=\{\left(X,Y\right)|X=\sum_{j=1}^{n}\lambda_{j}X_{j},Y\leq \sum_{j=1}^{n}\lambda_{j}Y_{j},\sum_{j=1}^{n}\lambda_{j}=1,\lambda_{j}\geq 0,j=1,\ldots ,n\}. \end{array}$$ (3)

Based on Eq (3), the determination of directional RTS and directional congestion proposed by Yang *et al.* [53], we applied the FDM method [55] to estimate the RTS on the right-hand and left-hand sides of efficient frontier *DMU*(*X*_0_, *Y*_0_) under the unit congestion effect by setting regions on the right and left sides of the efficient frontier *DMU*(*X*_0_, *Y*_0_). Model (4) is the RTS to the right side of *DMU*(*X*_0_, *Y*_0_), while model (5) is the RTS to the left side of *DMU*(*X*_0_, *Y*_0_).

$$\begin{array}{ccc} \text{max}\;\;\;\; & & \xi =\beta /t_{0}\\ s.t.\;\;\;\; & & \sum_{j=1}^{n}x_{ij}\lambda_{j}=\left(1+\omega_{i}t_{0}\right)x_{i0},i=1,\ldots ,m\\ & & \sum_{j=1}^{n}y_{j}\lambda_{j}\geq \left(1+\beta \right)y_{0},\\ & & \sum_{j=1}^{n}\lambda_{j}=1,\lambda_{j},\beta \geq 0,j=1,\ldots ,n \end{array}$$ (4)

$$\begin{array}{ccc} \text{min}\;\;\;\; & & \psi =\beta /t_{0}\\ s.t.\;\;\;\; & & \sum_{j=1}^{n}x_{ij}\lambda_{j}=\left(1-\omega_{i}t_{0}\right)x_{i0},i=1,\ldots ,m\\ & & \sum_{j=1}^{n}y_{j}\lambda_{j}\geq \left(1-\beta \right)y_{0},\\ & & \sum_{j=1}^{n}\lambda_{j}=1,\lambda_{j},\beta \geq 0,j=1,\ldots ,n \end{array}$$ (5)

When (*ω*_1_, *ω*_2_, …, *ω*_*m*_)^*T*^ is the directional factor of input, $\sum_{i=1}^{n}\omega_{i}=1$ and *t*_0_, *β* are coefficients, the directional RTS of *DMU*(*X*_0_, *Y*_0_) is as follows:

1. If *ξ*(*X*_0_, *Y*_0_)>1 (*ψ*(*X*_0_, *Y*_0_)>1) holds, then increasing RTS (IRS) occurs on the left-hand (or right-hand) side of point *DMU*(*X*_0_, *Y*_0_) in the direction of (*ω*_1_, …, *ω*_*m*_)^*T*^ and (*δ*_1_, …, *δ*_*s*_)^*T*^;
2. If *ξ*(*X*_0_, *Y*_0_) = 1 (*ψ*(*X*_0_, *Y*_0_) = 1) holds, then constant RTS (CRS) occurs on the left-hand (or right-hand) side of point *DMU*(*X*_0_, *Y*_0_) in the direction of (*ω*_1_, …, *ω*_*m*_)^*T*^ and (*δ*_1_, …, *δ*_*s*_)^*T*^;
3. If 0 < *ξ*(*X*_0_, *Y*_0_)<1 (0 < *ψ*(*X*_0_, *Y*_0_)<1) holds, then decreasing RTS (DRS) occurs on the left-hand (or right-hand) side of point *DMU*(*X*_0_, *Y*_0_) in the direction of (*ω*_1_, …, *ω*_*m*_)^*T*^ and (*δ*_1_, …, *δ*_*s*_)^*T*^;
4. If *ξ*(*X*_0_, *Y*_0_)<0 (*ψ*(*X*_0_, *Y*_0_)<0) holds, then congestion occurs on the left-hand (or right-hand) side of point *DMU*(*X*_0_, *Y*_0_) in the direction of (*ω*_1_, …, *ω*_*m*_)^*T*^ and (*δ*_1_, …, *δ*_*s*_)^*T*^.

The final directional RTS of *DMU*(*X*_0_, *Y*_0_) is determined by the RTS to both the left and right sides of *DMU*(*X*_0_, *Y*_0_). (1) When the left-hand side is IRS (DRS) and the right-hand side is also IRS (DRS), the RTS of *DMU*(*X*_0_, *Y*_0_) in the direction of (*ω*_1_, …, *ω*_*m*_)^*T*^ and (*δ*_1_, …, *δ*_*s*_)^*T*^ will be determined as IRS (DRS);

(2) When the left-hand side is IRS and the right-hand side is DRS, the RTS of *DMU*(*X*_0_, *Y*_0_) in the direction of (*ω*_1_, …, *ω*_*m*_)^*T*^ and (*δ*_1_, …, *δ*_*s*_)^*T*^ will be determined as CRS, which is the most productive scale size (MPSS);

(3) When either the left-hand or the right-hand side is congested, the RTS of *DMU*(*X*_0_, *Y*_0_) in the direction of (*ω*_1_, …, *ω*_*m*_)^*T*^ and (*δ*_1_, …, *δ*_*s*_)^*T*^ will be determined as congested.

The above studies all focus on determination of the directional RTS and directional congestion of the efficient frontier *DMU*(*X*_0_, *Y*_0_). To determine the internal directional RTS and directional congestion of *DMU*(*X*_0_, *Y*_0_), projection onto the frontier is required. The projection is performed as follows based on radial measurement Wei and Yan (2004) [58]:

$$\begin{array}{c} \left\{ \begin{array}{c} {\tilde{x}}_{i0}\leftarrow x_{i0},i=1,\ldots ,m\\ {\tilde{y}}_{0}\leftarrow \eta ⋆y_{0}, \end{array}\right. \end{array}$$ (6)

### Optimal input direction and the most productive scale size

To avoid choosing the direction randomly in the empirical research of the directional DEA model [53], combining with other optimization methods [60, 61], we develop a method to determine the optimal input direction and input region. We focus on the change in the directional RTS under congestion of the NSFC. As the definition of congestion above, congestion is a special return to scale (Wei and Yan, 2004) [58] and such inefficiency in the input-output system must be avoided. Thus, the optimal input direction is the direction with the lowest congestion rate in this paper, which is the proportion of congested projects to total projects.

First, we determine the relatively optimal input direction. The RTS of DMU(*X*_0_, *Y*_0_) varies in different directions. Therefore, it is critical to determine the relatively optimal input direction first before determining the RTS. Based on FDM (Rosen *et al.*, 1998 [55], Golany and Yu, 1997 [41]), taking the two input indicators in this study as an example, we select 21 directions every 0.1 in the region *ω*_1_ ∈ [0, 2], *ω*_2_ ∈ [0, 2], *ω*_1_ + *ω*_2_ = 2. We use *linprog* function in MATLAB to solve this linear programming problem based on model (4) and model (5), then we can determine the RTS of DMUs in each direction. Finally, we calculate the proportion of congested DMUs to the total DMUs.

Second, we determine the optimal input direction. Based on the proportion of congestion all directions, we use *polyfit* function in MATLAB to obtain the fitting curve of the proportion of congested DMUs depending on direction with minimum variance, and the lowest congestion rate region on the curve corresponds to the optimal input direction.

Finally, we determine the most productive scale size. According to the analysis of the first two steps, we insert the optimal input direction into model (4) and model (5) to calculate the RTS in this direction. Then the most productive scale size is the input of CRS in the optimal input direction.

## Empirical analysis about general program of the NSFC

### Input and output indicators

The General Program of the NSFC is essentially an input-output process. In this paper, we refer to the succinct indicators applied by Huang *et al.* (2016) [62], Bloch *et al.* (2016) [16], Shao *et al.* (2018) [63] and Albert (2020) [64] to analyze the directional RTS for the General Program of the NSFC. These indicators are as follows: (1) two input indicators: grant size and team size. Grant size is the funding provided by the General Program and team size reflects the human resource input. (2) Two output indicators: we use bibliometrics indicators to define two dimensions: quantity and quality. Quantity reflects the number of publications, while quality represents total citations.

### Data

In 2011, the NSFC announced a major upgrade of funding by the General Program by substantially increasing both the amount and duration of funding. These changes led to the grant size per program nearly doubling that year. We focus on whether larger grant size is better and whether the current grant size is the most productive. We selected data from three example disciplines physics, geography and management in 2011 to analyze and verify the change in directional RTS and the most productive input scale size. The three samples are the Department of Mathematical and Physical Sciences, the Department of Earth Sciences and the Department of Management Sciences, namely, physics, which that relies on experimental instruments for basic research, geography, which focuses on field exploration, and management, which emphasizes empirical investigations, under the condition of congestion.

The grant size input data come from receipts of the NSFC, and the team size input data come from the proposals. Output data are extracted from the search results based on the grant number in the Web of Science database (because a lot of publications of management are in Chinese, the output data is also from China national knowledge infrastructure (CNKI)). The deadline data denote the end of the project, which is December 2015. Moreover, to ensure the relevance between the publication output and the research theme of the project, the search criteria we used were “project number + participants + keywords (including one or more participants and one or more keywords)”. The detailed input and output data of three disciplines are shown in Table 1. Therefore, based on the above search criteria and after excluding projects without any output, the numbers of projects selected for directional DEA calculation were 308 from physics, 275 from geography and 156 from management.

**Table 1:** Input and output data of three disciplines in 2011.

Discipline	indicators	mean	median	mode	maximum	minimum	stddev
Physics	grant size	65.83	65	60	76	45	7.62
	team size	7.13	7	7	10	2	7.36
	publications	7.95	6	3	50	1	7.64
	total cites	59.49	22	1	762	1	99.67
Geography	grant size	64.82	65	65	85	48	9.01
	team size	7.85	8	8	10	5	6.06
	publications	4.55	3	2	20	1	3.74
	total cites	22.85	11	1	196	1	30.37
Management	grant size	42.01	42.03	42	50	32.2	2.37
	team size	8.52	9	9	14	3	8.06
	publications	5.22	4	2	28	1	4.18
	total cites	14.44	8	1	112	1	18.31

### Empirical analysis

Due to the large number of DMUs and space limitations, it is not possible to present the results of all DMUs. Therefore, this paper presents the RTS in proportion, and the complete results are available upon request. In model (4) and model (5), *ω*_1_ represents the direction of grant size input; *ω*_2_ represents the direction of team size input. Constraint conditions satisfy *ω*_1_ + *ω*_2_ = 1 and *ω*_1_ ∈ [0, 2], *ω*_2_ ∈ [0, 2]. For example, the direction of (*ω*_1_ = 0, *ω*_2_ = 2) represents a change in only team size with the grant size remaining constant, and the direction of (*ω*_1_ = 2, *ω*_2_ = 0) represents a change in grant size only with team size remaining constant. The other directions are defined similarly.

**First, we determine the optimal direction with the lowest congestion rate**.

**Fig 1:**
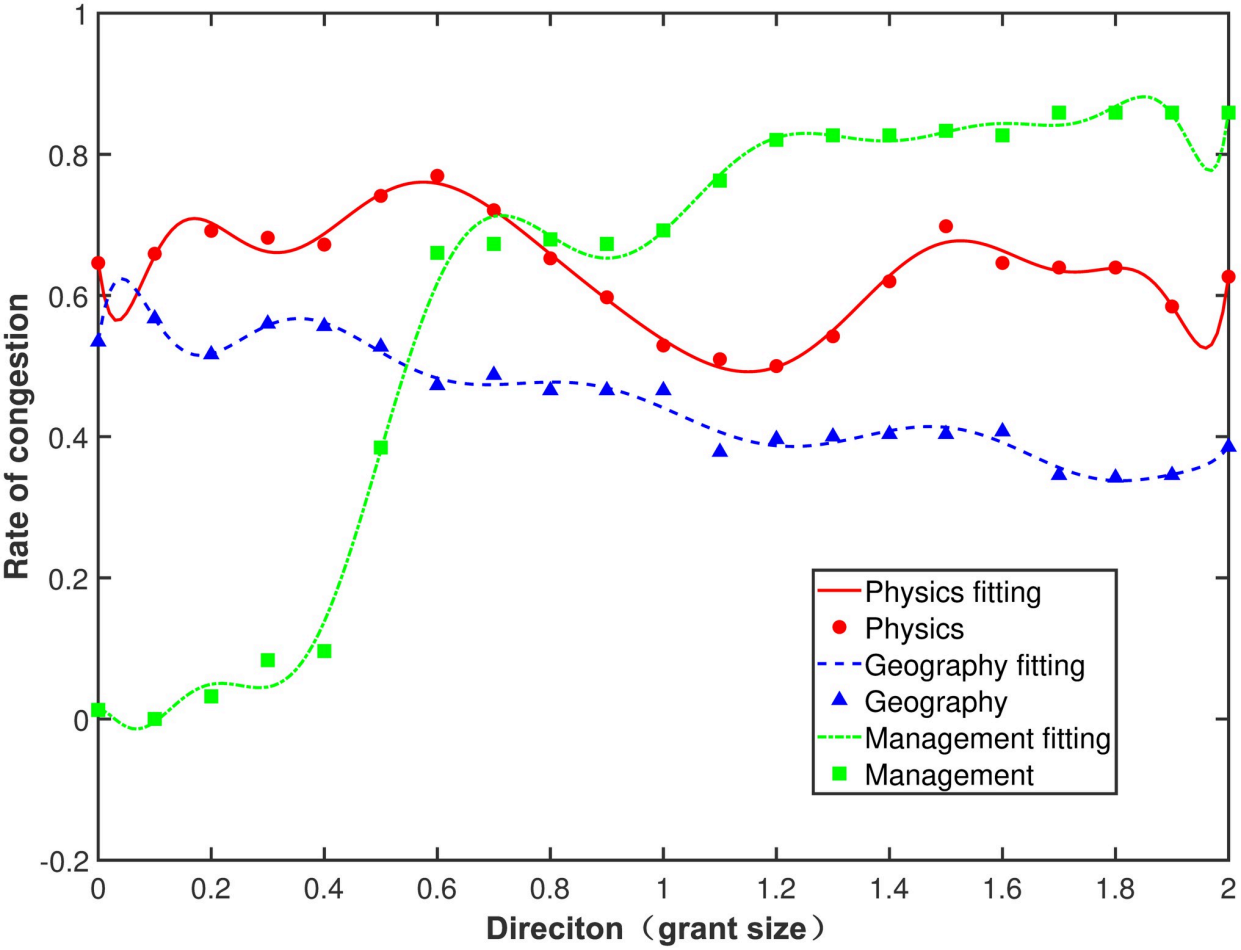
The rate of congestion in all directions of grant size (output: Publications).

**Fig 2:**
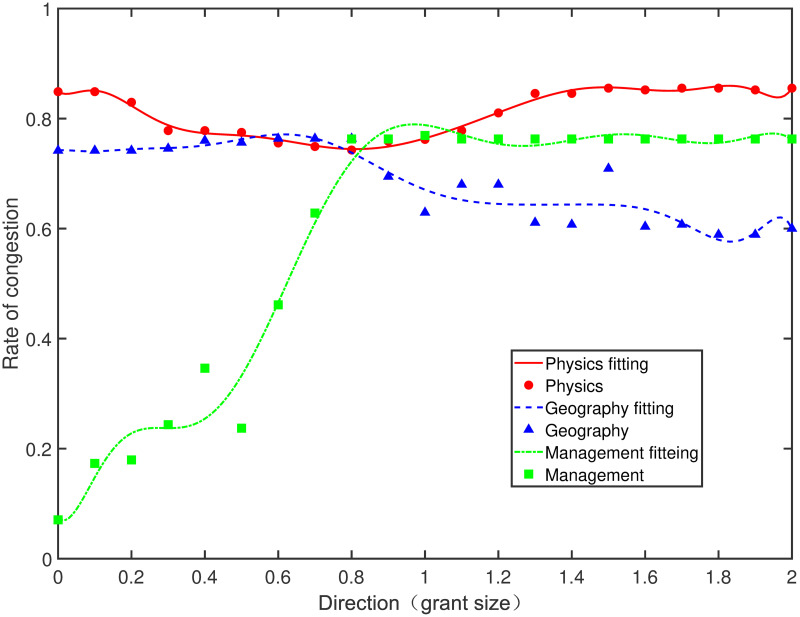
The rate of congestion in all directions of grant size (output: Total cites).

We choose one direction every 0.1 in the region *ω*_1_ ∈ [0, 2], *ω*_2_ ∈ [0, 2], and *ω*_1_ + *ω*_2_ = 1 for a total of 21 directions. After calculating the congestion rate of each direction, we fit the curve of the congestion rate to predict its extreme value range. As shown in Figs 1 and 2 the lowest rate of congestion is the optimal input direction.

When the output is set as publications, the congestion rates as a function of the direction of the three disciplines are shown in Fig 1. As indicated by the red solid line in Fig 1, physics has a high congestion rate in both ending directions of funding size. In the range of [1.0, 1.3], where *ω*_1_ ∈ [1.0, 1.3], *ω*_2_ ∈ [0.7, 1.0], and *ω*_1_ + *ω*_2_ = 1, the congestion rate is the lowest, and the direction where two input indicators change proportionally is the optimal input direction for physics. As indicated by the blue dashed line in Fig 1, the curve of the congestion rate of geography is the flattest and decreases gradually from left to right. In the range of [1.7, 2.0] for grant size direction, where *ω*_1_ ∈ [1.7, 2.0], *ω*_2_ ∈ [0.0, 0.3], and *ω*_1_ + *ω*_2_ = 1, the congestion rate is the lowest. The optimal input direction for geography is the direction where grant size changes while team size remains constant. As indicated by the green dash-dotted line in Fig 1, the curve of the congestion rate of management is the most fluctuating, going upwards from left to right. In the range of [0.0, 0.3] for grant size direction, where *ω*_1_ ∈ [0.0, 0.3], *ω*_2_ ∈ [0, 0.3], and *ω*_1_ + *ω*_2_ = 1, the congestion rate is the lowest, and the optimal input direction of management is to change team size while keeping grant size constant. When the output is publications, geography has the lowest congestion rate, and physics has a higher congestion rate.

Fig 2 shows the congestion rates as a function of direction for the three disciplines when the output is total citations. In general, the trend in Fig 2 is consistent with that in Fig 1, which indicates that the changing trend of the congestion rate with respect to the number of publications and total citations of each discipline is consistent. The congestion rate of physics (red solid line in Fig 2) is lower in the middle direction, that of geography (blue dashed line in Fig 2) is lower in the right direction, and that of management (green dash-dotted line in Fig 2) is lower in the left direction. In the range of [0.7, 1.2], where *ω*_1_ ∈ [0.7, 1.2], *ω*_2_ ∈ [0.8, 1.3], and *ω*_1_ + *ω*_2_ = 1 is the lowest congestion rate, the direction where two input indicators change proportionally is the optimal input direction for physics. In the range of [1.6, 2.0] for grant size direction, where *ω*_1_ ∈ [1.6, 2.0], *ω*_2_ ∈ [0.0, 0.4], and *ω*_1_ + *ω*_2_ = 1 is in the lowest congestion rate for geography. Thus, the optimal input direction of geography is the direction where grant size is changing while team size remains constant. In the range of [0.0, 0.3] for grant size direction, where *ω*_1_ ∈ [0.0, 0.3], *ω*_2_ ∈ [1.7, 2.0], and *ω*_1_ + *ω*_2_ = 1 is the lowest congestion rate of management. The optimal input direction of management is to change team size while keeping grant size constant. The overall congestion rate of the three disciplines is higher when the output is total citations rather than publications.

**Second, we determine the RTS distribution in the optimal input direction**.

The optimal input directions of physics, geography and management determined in last step are brought into model (4) and model (5). For physics, the proportions of IRS, CRS and DRS are 12.01%, 9.09% and 28.90% under the optimal input direction (*ω*_1_ = 1.2, *ω*_2_ = 0.8) with publications as the output. In the optimal input direction (*ω*_1_ = 0.8, *ω*_2_ = 1.2) with total citations as the output, the proportions of IRS, CRS and DRS are 13.51%, 8.68% and3.54%. Notably, even in the optimal input direction, the congestion rate of physics still exceeds 50%.

For geography, the proportions of IRS, CRS and DRS are 44.36%, 11.64% and 9.45% under the optimal input direction (*ω*_1_ = 1.9, *ω*_2_ = 0.1) with publications as the output. In the optimal input direction (*ω*_1_ = 1.6, *ω*_2_ = 0.4), the proportions of IRS and CRS are 33.45% and 6.18%. The rate of congestion with publications as the output is lower than that with citations as the output. The optimal input direction of management with the two output indicators is the same, i.e., (*ω*_1_ = 0.0, *ω*_2_ = 2.0), with changing team size and constant grant size. In this direction, the proportion of CRS with the two outputs is 1.28% and 12.82%. However, the proportion of IRS exceeds 65%, which shows that a higher input in this direction can easily result in a higher output for management.

**Finally, we determine the most productive scale size in the optimal input direction**.

We can obtain CRS under the optimal direction of physics, geography, management. Then, we analyze the DMUs in CRS, and insert its input distribution interval as the most productive scale size, as shown in Table 2. By comparing the most productive scale size with the actual grant size, we can help decision-makers to determine whether the grant size is increasing or decreasing.

**Table 2:** The optimal investment interval of the general program.

Discipline	Input	Actual funding in 2011		DEA results			
				Output: Publications		Output: Total citations	
		Mean	Range	Mean	Range	Mean	Range
Physics	Grant size	65.83	[60, 65]	59.93	[59, 61]	60	[59, 60]
	Team size	7.13	[5, 8]	4.04	[3, 5]	4.29	[3, 5]
Geography	Grant size	64.82	[60, 70]	65.69	[60, 70]	71.25	[65, 80]
	Team size	7.85	[6, 9]	7.54	[7, 8]	6.75	[6, 7]
Management	Grant size	42.01	[40, 45]	38.00	[38, 38]	41.02	[40, 44]
	Team size	8.52	[8, 10]	9.00	[9, 10]	9.75	[9, 11]

For physics, the most productive scale of the grant size input under the optimal direction is concentrated in the range [58, 61] (in units of ten thousand yuan, the same as the following), which was lower than the actual average grant size of 65.83 in 2011. The most productive scale of the input team size under this direction was concentrated in the range [3, 5] (in units of person), lower than the actual average team size of 7.13 in 2011. In terms of the optimal direction of physics, where the two input indicators change proportionally, on the basis of both the quantity indicator and quality indicator, the grant size and team size inputs should not be further increased: other management measures should be taken to improve the performance of the project team.

The actual average grant size of geography was 64.82. We calculated the most productive grant size input to be 65.69 (output: publications) and 71.25 (output: total citations), slightly higher than the actual grant size. The actual team size in 2011 was concentrated in the range [6, 9], slightly higher than the most productive scale size calculated by DEA. For geography, when the output is the quantity indicator, the input in 2011 is close to the relatively most productive scale size; when the output is the quality indicator, the grant size can be further increased.

The actual average grant size of management was 42.01. We calculated the most productive grant size input to be 38 (output: publications) and 41.02 (output: total citations), slightly lower than the actual grant size. The actual team size in 2011 concentrated in the range [8, 10], slightly lower than the most productive scale size calculated by DEA. For management, increasing team size would further improve the performance.

## Conclusion

With the current trend towards larger grants, whether larger grants foster ‘‘excellence’’ is an important question. A general result for research grant size is that there exists a critical mass threshold but no empirical evidence of RTS. Thus, we focus on whether current grant sizes are the most productive by studying the RTS of NSFC’s General Program. Based on the grants of NSFC’s General Program, this paper studies the change in directional RTS in the presence of congestion, constructs a directional DEA model under congestion, and conducts an empirical study.

We selected three samples of data of physics, geography and management in 2011 to analyze and verify the change in directional RTS and the most productive input scale size. In this paper, we consider 21 directions with intervals of 0.1 according to grant size and team size. To avoid congestion, we take the direction with the lowest rate of congestion as the optimal input direction. Based on the above results, the change in RTS is calculated with directional DEA model, and the most productive scale size is determined according to the grant size in CRS. The optimal input direction with the lowest rate of congestion and the most productive scale size are important information for project management.

Moreover, the practical analysis proves that (1) the three disciplines showed congestion effect in all directions; overall geography has the lowest rate of congestion. Furthermore, the proportion of congestion among three disciplines with publications as the output is higher than that with total citations as the output. (2) The optimal direction of inputs differs for the three disciplines. The optimal input direction of physics is where the proportional change occurs between team size and grant size, i.e., the team size increases with increasing grant size. The optimal input direction of geography is the direction where grant size increases while team size remains constant. The optimal input direction of management is the direction where team size increases while grant size remains constant. (3) The optimal input calculated via the directional DEA model is different from the actual grant size in 2011 for the three disciplines. For physics, the most productive size of the two inputs under the optimal direction was lower than the actual average of the two inputs. The most productive grant size was higher than the actual average grant size, but the optimal team size was lower for geography. By contrast, for management, the most productive grant size was lower than the actual average grant size, but the optimal team size was higher. A larger grant size does not necessarily increase productivity; this confirms Dimke‘s (2019) [23] result that most surveyed scientists prefer small- or medium-sized grants to pursue their ideas and advance their scientific careers.

Based on the above research, suggestions can be provided for future science fund managers: First, there are differences in the most productive scale size of inputs of NSFC program in different disciplines. Thus, when adjusting NSFC’s grant size, one need pay attention to the differences among disciplines in the future, and distinguish the reasonable funding range of different disciplines, e.g. based on the empirical study, the current grant size of physics should not be increased, and other management measures should be taken to improve performance. The grant size can be further increased to improve performance for geography. However, the grant size of management should not be further expanded, but increasing the team size can significantly improve performance. Second, in order to reasonably allocate fund resources, NSFC should not only consider the existing development planning and strategic demand orientation, but also refer to the funding performance of different disciplines, so as to further improve the funding performance of NSFC. Finally, for the grant size, NSFC needs to strengthen the application requirements of the project leader on demand and the control role of the review experts in the review process.

## Supporting information

S1 File Source data supporting for empirical analysis sections. (XLSX)
